# 3D printed extraction devices in the analytical laboratory—a case study of Soxhlet extraction

**DOI:** 10.1007/s00216-021-03406-4

**Published:** 2021-05-27

**Authors:** David J. Cocovi-Solberg, Manuel Miró

**Affiliations:** 1 Institute of Analytical Chemistry, Department of Chemistry, University of Natural Resources and Life Sciences in Vienna, BOKU, Muthgasse 18, 1190 Vienna, Austria; 2 FI-TRACE group, Chemistry Department, University of the Balearic Islands, Carretera de Valldemossa km 7.5, 07122 Palma de Mallorca, Spain

## Introduction

3D printing was introduced in the 1980s but only now has gained widespread acceptance among practitioners because of (i) the friendlier software interfaces for digital computer-assisted designs (CAD), and the ensuing computer-assisted manufacturing (CAM) of the 3D object; (ii) availability of low-cost custom-grade printers; and (iii) the advantages that present against milling or other classical subtractive technologies, that is, enables complicated geometries to be easily designed in a single step, generates minimum residues, and allows fast prototyping. Chemists have followed this trend and incorporated 3D printers in their research laboratories and lecture halls, demonstrating the possibilities of this technique in a plethora of academic publications [1–3].

Notwithstanding the versatility of the printed educational resources, their applicability in the analytical chemical laboratory has not yet attracted much attention because of the limited chemical compatibility [4–8] of the majority of polymeric 3D prints: Their chemistry is polar (methacrylate-epoxy, polylactic acid, nylon, and acrylonitrile butadiene styrene), and thus, solvents, acids, or aggressive reagents put the stability of the prints in jeopardy. This may be the reason why most of the applications found in the chemistry literature resort to visualization of tridimensional structures [1, 9, 10], fabrication of non-wetted mechanical supports [11, 12], or disposable microfluidic chips [7, 13] that can merely perform a handful of unitary processes before the wetted surface gets degraded or some chemical components of the process sorb onto it irreversibly. A key player remains hidden in the shadows of this complex landscape: the 3D printed polar objects are superbly compatible with the majority of non-polar solvents [5, 14] and those fabricated with stereolithography (SLA) are also watertight and transparent, a triple-win situation in the teaching domain. Low-cost SLA printers of less than 200 € are available, work without need of a computer connection [15], have a minimal footprint, and the only safety concerns are the minimally irritant resin and volatile components in the course of the printing process.

Given the potential of 3D printing and the leading role that chemists will play in its development, the presentation of this technique to the young generation of chemists at the undergraduate level is not only capital but also fostered by the new guidelines of European Higher Education Area [16, 17] as a transversal and interactive competence. Aspects that might become pivotal in the development of methods for analytical chemists are (i) the fast, transparent, and watertight 3D printed prototypes, (ii) their chemical compatibility that must be carefully assessed for the different analytical applications, (iii) the reversed phase or cation exchange capabilities of the polymers [4], and (iv) the possibility that the uncured resin components contaminate the sample by leaching monomers and oligomers.

The solid-liquid extraction of hydrophobic compounds from environmental solids is an analytical application very well suited to the analytical chemistry laboratory in which 3D prints can be leveraged because it avoids light eluotropic solvents and very high temperatures. To this end, we herein introduce a laboratory exercise for the “Integrated Analytical Chemistry Laboratory” (last semester of the BSc in Chemistry (year 4) at the University of the Balearic Islands, Spain) that aims to exploit 3D printing in extraction technologies. The students will design and prototype their own Soxhlet extractor by SLA whereupon it will be employed in a sediment analysis workflow, by attaching it to standard glassware, such as an Erlenmeyer flask and Liebig condenser, for extraction of polycyclic aromatic hydrocarbons (PAHs) using hexane prior to high-performance liquid chromatographic (HPLC) analysis.

The abovementioned points referring to 3D printing (i to iv) will be implicit in the laboratory exercise, which will follow a (constrained) guided inquiry structure: The idea behind it is to engage the students in elucidating the possibility of replacing the standard glassware for low-cost, tailorable 3D prints. The students are expected to learn the CAD/CAM software, fabricate the extractor, and compare the performance with a glass counterpart, and even with a third extraction approach for validation purposes. The benefits of customization will be exemplified by designing a smaller working Soxhlet that permits less solvent and energy consumption than conventional counterparts and can be fabricated in 2 days. The disadvantages will be found when the addition of ≥5% acetone as additive to the extractant is proven incompatible with the resin, or the boiling temperature of the toluene is too high for use with the 3D resin. Detection and quantification of fluoranthene will be compromised when the unspecific leachates of the resin overlap with that analyte in the chromatogram. Finally, all the abovementioned points will have to be explained by the students from the chemical point of view (molecular interactions, partitioning, kinetics, heat and mechanical properties or material science) in their final report in which the pivotal position of chemistry in the developments of this still evolving tool will be demonstrated.

## Session I: CAD of the Soxhlet extractor

The first session of this exercise (4 h) is organized in a separate computer classroom. Prior to starting, the students are provided with two previous papers from the authors describing the use of 3D printing in the analytical laboratory [4], and the role of 3D printing in analytical extraction workflows [3] as a background. The 16 students attending the course are asked to design a Soxhlet model with a CAD software. Usually, they perform all the tasks in groups of 2 individuals, but for this first session, two groups (4 students) will work together. We use the freeware 123D Design (Autodesk, San Rafael, CA, USA) for the sake of simplicity and clean interface.

During the first hour, the students are trained by the instructor or a PhD student on the variety of standard operations enabled by the software [18]. The teacher or PhD assistant demonstrates how to insert a simple three-dimensional body, as a cube, sphere, or cone to name a few, and immediately passes the control to the student for replicating the action until all of them insert a body. Then the teacher demonstrates some of the “manipulation” tools needed to model the object, namely, extruding, revolving, snapping, merging, subtracting, and filleting. Afterwards, the students practice several of the actions and commands taught by the teacher. For example, the instructor draws a whole object on its own as, e.g., a house [18]. A cube is inserted as a main body, a triangular prism is snapped as a roof, a stretched cube will be a chimney, and the windows are the voids subtracted by cubes. The students as a group must replicate the house. The teacher challenges them, asking for further modifications as, e.g., different sizes, varied shapes, a porch, more doors, a Vantongerloo-inspired sculpture in the garden, or the Christmas tree of the last winter. It is explicit that all of them must participate equally. The “house” exercise increases their independence with the software prior to the main lab exercise encompassing CAD modeling.

After this first hour, a glass Soxhlet body is given to the students and they are asked to model it with a reduced inner volume. To foster the guided inquiry model, a broken Soxhlet can be given as well and ask them to fabricate a replacement for the second part of the exercise. In our case, the justification of the exercise is to compare whether the 3D printed labware can substitute the conventional glassware. In some instances, a given student had previous experience with CAD software as a hobby or attendance of freely configurable subjects as “technical drawing.” In those cases, the group required less attention, and this particular student was given the duty to assess that all of the individuals within the team commanded the software at equal times, in an example of 2-phase collaborative learning [19]. They are prompted to break the model down into simple geometries and insert them in the software at appropriate places. At this point, they needed minimum supervision, usually every 20 to 30 min.

**Fig. 1:**
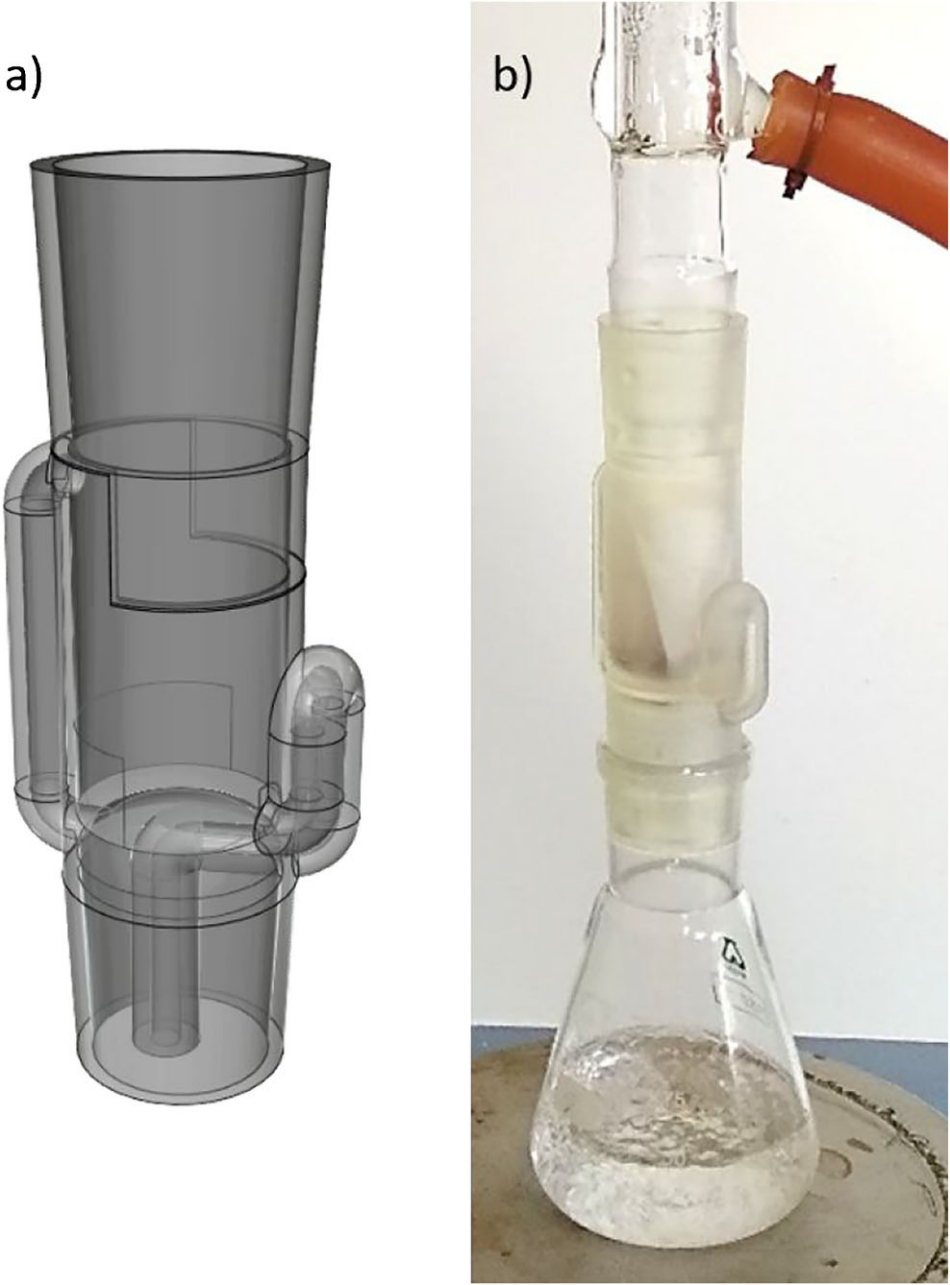
**a** Digital model of the Soxhlet extractor, showing the central body, the top and bottom 29–32 tapers, the distillation path (left), and the syphon arm (right). **b** Printed 3D piece working with off-the-shelf components: Erlenmeyer flask and Liebig condenser

We realized that most of the groups invested too much time in small morphological features that have no practical relevance, so we decided, in some instances, to provide them with the Soxhlet CAD model from the instructor (Fig. 1) after initial unsuccessful trials (usually after 60 min). Tapers were usually not drawn correctly, and the instructor had to assess the measures during the exercise. Students are advised to search on the Internet for the rationale behind the 29/32 tapper number, and then suggested to make it by CAD by inserting a cone with a 29-mm diameter basis, the appropriate height to get the 1:10 conicity, and then truncate the cone to the 32-mm height. Some details that assure a good print are included as a supplementary information and should be introduced to the students. If the model is acceptable, it could be printed, otherwise and if the students wanted to ameliorate it, they could do it as homework in exchange of extra points for the qualification.

The first students’ Soxhlet printed did not work out because the syphon was blocked, and this was difficult to observe in the CAD model. Therefore, this group used the 3D printed Soxhlet provided by the instructor (see SI for specific details of the CAD design). Other problems experienced during the authors’ previous trials were the gargling effect in the syphon if the inner diameters were not big enough, and the excess of resin consumption. Because of time constraints, we suggest that unless the students’ prototype will obviously work, students print the model designed by the instructor (Fig. 1; the .stl file is given in SI for replication by interested readers). When we justified the reasons for which the students’ prototype would not work or would be difficult to print, as well as the time constraints, the students showed the same engagement with the provided CAD model as that of the group that did print their own. All the groups wanted to take their Soxhlet home as a “reward.” This was allowed since the overall price was below 8€ (see SI), and the printing of a new Soxhlet per group is deemed beneficial for confronting the black box perception of some students about 3D printing [20].

In the last hour of this first practical session, the instructor introduces the concept of CAM software as the one used for controlling the 3D printer (in our case Preform for Form2 SLA printer). The importance of moving, rotating, and adding supports to the CAD model is explained. Briefly, the move action prevents the laser from degrading a section of the silicon film of the tank prematurely; tilting the model prevents failures due to strong overhangs, and the addition of supports allows printing those that are unavoidable, as well as separating the model from the platform, since the first layers are usually printed with increased exposure time for enhanced adherence to the printing platform. The model is afterwards loaded in the printer and a brief description of the working principle is presented. UV polymerization is also linked to organic chemistry subjects.

Our model required 7 h and 42 min to get printed, and thus could not be done during the duration of the laboratory session. We decided to start printing in the morning, so the students from that group could see the end of the printing during the laboratory session. The starting time was negotiated with the students, so if they wanted, they could be present in the first minutes of the print. This attendance was not mandatory, but for three out of the four groups, all the members were available. At the end of the print, the almost finished extractor can be seen entering repeatedly into the resin tank, as well as the UV laser scanning the different layers, which is much more stimulating for the students than the initial printing hours, when the operation of the printer cannot be easily understood. Note that the orange cover of SLA printers prevents eye damage by the UV laser. Afterwards, the instructor retrieves the print from the moving platform followed by cleaning in an isopropanol bath and curing in a UV oven for 1 day (see SI for further details). Students are required to watch the cleaning process. Note that it is not possible to use the Soxhlet until 2 days after the design, so this exercise must be interleaved with another lab exercise, depending on the practicum schedule.

## Session II: Application to real samples

In the second session (4 h), the students (i) assemble the Soxhlet extraction system, (ii) rinse it with appropriate solvent pending use, (iii) perform the solid-liquid extraction of PAHs from a real environmental solid sample, and (iv) analyze the extracts and process the chromatographic data. Two students work with the 3D printed Soxhlet and two with the glass counterpart in parallel. During the unattended Soxhlet extraction of the solid material, students perform together an ultrasound-assisted extraction using a probe system with the aim of introducing the concept of validation resorting to a different extraction approach, that is, the results from the 3D printed Soxhlet and the glass counterparts might be statistically identical, but different from another extraction methodology.

The low-cost extraction system is assembled with the 3D printed Soxhlet body and off-the-shelf components: a 30-cm Liebig condenser, a 100-mL Erlenmeyer flask, and a heating plate. The conventional extraction system is assembled with a Soxhlet body, Dimroth condenser, round-bottom flask, and electric mantle. A thimble is created by wrapping a piece of laboratory filter paper, which is introduced into the Soxhlet body. Hexane is selected as the extraction medium instead of other common mixtures, such as hexane-acetone or toluene because of the greater hydrophobicity and lower boiling point, respectively. The solvent reservoir is filled with 2 syphoning volumes (ca. 20 mL and 60 mL, respectively) of hexane through the Soxhlet and the system (without sample) is run for 30 min (12 and 2 cycles, respectively) for preventing carryover or unspecific interfering compounds leached from the printed material. A suspension of white flakes can be observed in the reservoir of the low-cost setup and is attributed to the reactivity of the solvent at increasing temperatures toward the still-green 3D printed module but does not jeopardize further applicability. Those first volumes are exchanged with fresh hexane, the thimbles filled with 1.00 g of PAH-containing sediment (see SI for further information as to the real sample), and the heating elements turned on again. After 2 h of operation, the 3D printed and glass Soxhlet systems are stopped, the bodies detached from the flasks, and the latter kept on the heating elements for gentle drying of the extracts in the fume hood. Students may require help for disassembling the heated apparatus without exposing their skin to the hexane vapors (see SI for comments on the differential thermal expansion of glass and resin). When the flasks are cooled at room temperature, the residues are reconstituted in 5 mL of acetonitrile, recovered with a glass syringe, filtered through a 0.45-μm Nylon filter, and made up with acetonitrile to 10 mL using a volumetric flask.

**Fig. 2:**
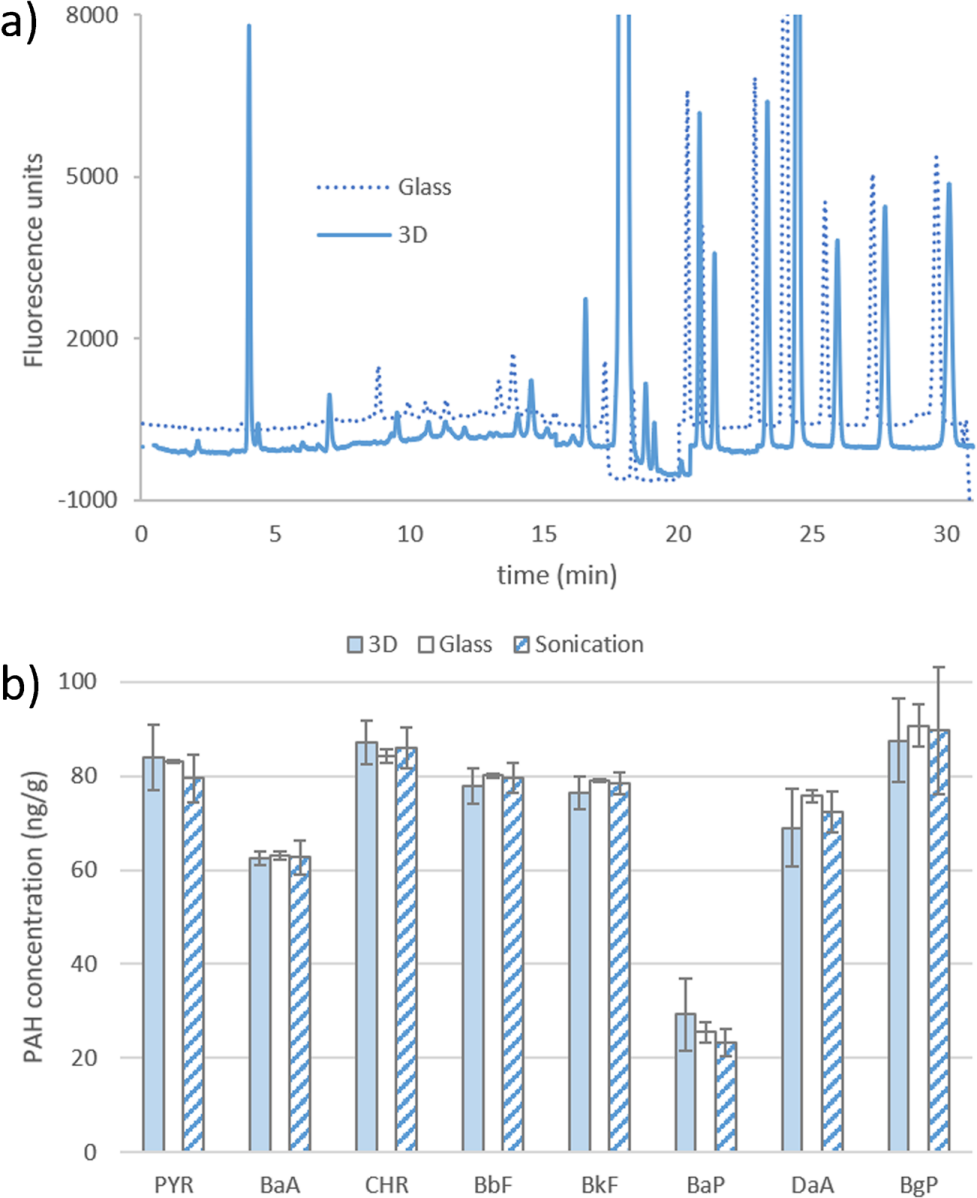
**a** Overlap of chromatograms of PAHs in sediment extracts obtained by the commercial glass Soxhlet (dotted trace) and 3D printed extractor with off-the-shelf configuration (solid trace). The glass trace has been shifted 400 FU and 30 s for ease of comparison. Ghost peaks come from the 3D print, but only that at 18.2 min interferes with the analytical measurements. **b** Comparison of the concentrations of PAH in the assayed sediment by three different extraction methods: 3D printed Soxhlet, classical glass Soxhlet, and ultrasound-assisted acetonitrile extraction. Abbreviations for PAH: PYR (pyrene), BaA (benz[a]anthracene), CHR (chrysene), BbF (benzo[b]fluoranthene), BkF (benzo[k]fluoranthene), BaP (benzo[a]pyrene), BgP (benzo[g,h,i]perylene), DaA (dibenz[a,h]anthracene)

While both Soxhlet systems are running, the students complete the ultrasonic probe-assisted extraction: 1.00 g of sediment is introduced in a 50-mL beaker along with 10 mL of acetonitrile and sonicated for 5 min. The suspension is recovered and filtrated through a 0.45-μm nylon filter with a 10-mL glass syringe and made up with acetonitrile to 10 mL. Thereafter, the students start equilibrating the HPLC column and analyze a standard solution. HPLC theory and applicability is taught in other lectures in analytical chemistry and instrumental analysis and lab courses as well including “Experimentation in analytical chemistry” (6th semester) or “Advanced chemistry laboratory” (7th semester), so students have an active role, yet always supervised by a technician. The guided inquiry format is respected as far as the time constraints enable: the students must scrutinize available literature and national/international norms to decide whether a standard method for PAHs is available or rather they must create their own. Only one 100 μg L^−1^ standard is injected by the students, which is already prepared (time constraints). A 5-level calibration curve is provided afterwards for data interpolation. HPLC conditions used in this work are available in SI. Examples of chromatograms for the 3D printed and classical glass Soxhlet are shown in Fig. 2. Four artifact peaks are detected in the extract from the 3D print but not in the glass one and are thus attributed to the contribution of unpolymerized monomers or oligomers leached along the PAHs from the sediment. In our case, only the artifact at 18.2 min is interfering with the HPLC analysis by overlapping with the fluoranthene peak, because of having the same retention time, eliciting fluorescence response at the same wavelength pair than the analyte and being much more concentrated. The concentrations of individual PAHs extracted from the sediment revealed no significant statistical differences between the three methods for any of the analytes (Fig. 2b). This demonstrates that the 3D printed Soxhlet yields the same results as the glass counterpart and ultrasound-assisted extraction, and that those results are not subjected to a bias inherent to the Soxhlet extraction.

## Students’ assessment

After completing the two lab sessions, the students must fill an online questionnaire based on convergent, evaluative, and multiple-choice questions that was designed as a formative assessment tool to evaluate the achievement of the aims proposed in the introduction and the students’ ability of connecting 3D printing with other subjects of the chemistry syllabus. We paid special attention to introduce in the laboratory exercises all the concepts that would be asked for in the questionnaire. Critical thinking is expected in order to complete the post practicum questionary, such as not to neglect the power consumption of the printer to evaluate costs of the 3D printed Soxhlet, recording the syphoning frequency of both Soxhlet apparatus for comparing their energetic efficiency or observing the need to retighten the 3D printed system during warming, to name a few. To guide students toward the correct answers, they could see the questions previously and check literature  before submit the answers online. They had up to 3 attempts to answer all the questions, from which they receive a feedback every time. Yet, a point was subtracted from the final qualification in every second and third attempt. Final qualifications (0–10, the latter being the maximum) were 10 for 44% of the students, 9 for 31%, 8 for 13%, and 7 for 12%. In no case, the questionary lowered the grades of the students. The questionary was reviewed upon the application to other audiences (master, and PhD students); and thus, improved (and expanded) questionnaires were generated, from which a model test can be found in SI.

## Conclusions

Given the strong relationship between 3D printing and analytical chemistry, we have herein proposed an integrated laboratory practicum in which the students are engaged to design a Soxhlet extractor with CAD/CAM software followed by fabrication of the prototype using an inexpensive SLA 3D printer, for further application to the analysis of an environmental solid. The fast prototyping by 3D printing makes this proposal amenable to the students’ laboratory time and opens the door of 3D printing to educational applications as a cost-effective alternative to dedicated glassware because it (i) allows customization of volumes and shapes, (ii) yields transparent components readily compatible with grounded glassware, and (iii) enables integrating several functionalities in a single print. The results demonstrate that the prints are completely compatible with non-polar solvents at moderate temperatures. The extracts however might contain some interfering compounds resulting from leached monomers/oligomers, but the comparison against a classical glass Soxhlet and acetonitrile-based ultrasound-assisted extraction revealed no significant differences for most of the target analytes. The guided inquiry format of lab courses paved the way for introducing a hands-on exercise with the CAD/CAM software and 3D printing in the frame of analytical chemistry, and to relate the technical details of such manufacturing process with the underlying chemistry principles learned in previous semesters.

## Supplementary information

ESM 1 (DOCX 142 kb)
