# Supplementary material for: 3D printed extraction devices in the analytical laboratory—a case study of Soxhlet extraction
Source: Anal Bioanal Chem. 2021 May 27;413(17):4373–8. doi: 10.1007/s00216-021-03406-4 (PMC8245360; doi:10.1007/s00216-021-03406-4)
Supplement: Supplementary file 1 — (DOCX 142 kb) [file 216_2021_3406_MOESM1_ESM.docx]

# Supplementary Information

# 3D printed extraction devices in the analytical laboratory - A case study of Soxhlet extraction

David J. Cocovi-Solberg, Manuel Miró

## 3D printed Soxhlet design

*Volume of the Soxhlet body***.** One of the main advantages of the customized 3D printed Soxhlet extractor is that the volume can be minimized, which implies less solvent and sample consumption, and faster cycles. The students can design their own extractor or create the model of a real one and scaling it (not the tappers). In the design attached as SI, the central body has an inner diameter of 2.5 cm and thus, the inner volume of the body was ca. 10.5 mL (from the inner base to the upper saddle point of the siphon arm), while the smallest commercial glass counterpart was of 30 mL.

*Tapers***.** The tapers must meet the original size to fit within the glass components. The upper and lower conicities were designed to match standard 29/32 tapers, but custom designs to any other dimensions can be fabricated at will whenever the size of the complementary glassware is known in advance.

*Wall thickness***.** Walls of the 3D prints must be thicker than the glass originals for a successful and repeatable print. The reduced thermal conductivity of the printed resin (0.18 W m^-1^ K^-1^ for polymethyl methacrylate plastic) against the glass counterpart (ca. 1.2 W m^-1^ K^-1^ for 3.3 borosilicate glass) created an almost thermally isolated piece. The vapor generated in the solvent reservoir passes more easily through the distillation bypass in the printed part than for the glass counterpart, because in the latter, a significant amount of energy is spent in keeping the distillation bypass at high temperature. This also allows faster cycles. In the proposed design, the wall thickness was 2.5 mm through all the structure. It can be however easily re-optimized for minimal resin consumption.

*Arm dimensioning***.** Both arms have an inner diameter of 4 mm. The vapor pass has a length of 4 cm and the syphon of 2 cm. Dimensions should be strictly maintained for a smooth syphoning effect. Complications include a gagging and continuous syphon that prevents a discontinuous extraction, or that the pressure built in the flask rejects the syphoning and thus the liquid is retained within the intermediate compartment.

## Soxhlet fabrication by stereolithographic 3D printing

We recommend active participation and engagement of the students throughout the entire fabrication process as it will confront the black box perception of 3D printing [1]. Further the stereolithographic (SLA) resins require minimum safety measures (irritant) and low cost consumer-grade SLA printers have a small footprint and are able to work as standalone devices[2], which makes them readily available in the analytical chemistry lab.

In our case, the model was printed using a Form 2 printer (Formlabs, Sommerville, Massachusetts, US), FLGPCL04 clear resin and Preform CAM software (Formlabs). It was printed vertically and without supports, with the face that would connect to the solvent reservoir attached to the moving printing platform, and thus, the part shown on the bottom of Fig. 1 in the main text would be printed first. This configuration was selected because the tube from the syphon arm provided further anchorage to the printing platform. In this configuration, the printing process required 1131 layers and was completed in 7 h and 42 min, with a resin consumption of 29.55 mL and a total cost of 5.03 € of resin and 2.39 € of electrical power. As a comparison, the glass Soxhlet was acquired by 69.19 € from a low-cost provider.

The printing time is too long to allow complete printing during the lab exercise, so we propose that students will only participate in the start and end of the printing process. The post-printing steps are conducted by the instructor and involve (i) extract the print, (ii) immerse it in cleaning-quality isopropanol for 30 min for removing the non-polymerized resin remains on the print (mind the internal syphon), (iii) air dry the piece and (iv) cure it. In our case, we used an UV oven (KA-9180, PSKY, China) furnished with two 8W low-pressure mercury lamps for 24 h for post-curing of the SLA prints. This time can be reduced by resorting to a high pressure mercury lamp [3] or to high power UV LEDs matching the SLA laser wavelength, but the absorption of UV light by the external surfaces of the prints prevented the complete curation of the inner turns that could be alleviated by a real-time thermal curation process, as commented below.

The 3D printed device was used straight away after the curing procedure without sanding nor polishing and thus, the Soxhlet body was translucid rather than transparent, but allowed to visualize the whole extraction and syphoning cycles. For applications in which an easier visualization is required, a procedure for enhancing the transparency in expense of a longer fabrication time has been described elsewhere [3, 4]. In brief, the print is fine sanded, a further layer of liquid resin is added to the external surface, and the device is UV-cured again. To this end, flat external surfaces can facilitate the sanding procedure. Students could be asked to propose and explore the different design possibilities and post-processing procedures [5] depending on the scheduled laboratory sessions.

## Sample

In our case, the sample consisted of a marine sediment of sandy texture, sampled along the Mallorcan coastline, dried, fortified with 100 ng g^-1^ of the 16 EPA polycyclic aromatic hydrocarbons (PAH) in acetone, evaporated to dryness, moisture restored to 10% with addition of water and aged (in our case for 5 years) under biotic conditions in an amber jar for minimizing photodegradation.

## First use of the 3D printed Soxhlet extractor

The chemical reactivity of the “green” 3D printed extractor or temperature effects onto the prints as described in the main text occurred probably because the inner surfaces of the print (main body, distillation bypass and syphon arm) were not cured enough due to the power and geometry of the curation oven.

It is important to highlight that the roughness of the unpolished resin, along with the higher thermal expansion coefficient of the resin (87.2∙10^-6^ K^-1^[6]) compared to borosilicate glass (3.3∙10^-6^ K^-1^) led to a very tight seal of the male 3D printed piece against the female ground glass components, while the female 3D-printed connections loosened against the male glass components and had to be slightly retightened after heating. Contrariwise, the Liebig condenser had to be removed quickly after the sediment extraction procedure before the cooling start tightening the printed body against the condenser.

As indicated in the main text, the first blank extraction (without sample) conducted with a brand new 3D printed extractor and hexane yielded a white suspension rather than a clear organic solvent. The fact that those white flakes were not observed in further extractions suggests that an in-situ fast thermal rather than UV-triggered curation is occurring and this could explain the continuous heat flow of -100 µW K-1 from 50 to 300°C in differential scanning calorimetric curves reported for the same resin [3]. Previous applications of hot solvents using SLA prints reported a partial dissolution of the resin [7] that we did not detect with hexane. The difference in the observed behavior can be attributed to the varied temperatures in both applications (68°C in the current one vs 81°C in the literature), small differences in the resin composition and the use of a different solvent mixture. In the time of submission of this manuscript, the Soxhlet extractor was used more than 50 h with hexane as extractant without stress or fouling effects.

## HPLC Analysis

In our case, 10 µL of the acetonitrile reconstituted extract (see the main text) were injected without further treatment in a Jasco-4000 HPLC system (Jasco Inc, Maryland, US). The chromatographic column was a Pursuit 5 PAH (250 mm x 4.6 mm, 5 µm particle size) held at 30°C. The flow rate was 1 mL min^-1^. The gradient method started with 50% acetonitrile (mobile phase B) that was linearly increased to 70% (until min 13), and then 100 % (min 15), maintained until min 29, and then decreased to 50 % (min 31). Complementary mobile phase A was MilliQ Water (resistivity > 18.2 MΩ cm). The excitation and emission wavelengths of the fluorescence detector were set to 275 nm and 350 nm respectively, but changed to 260/420 (min 15), 270/440 (min 17.1), 260/420 (min 20), 290/430 (min 22.5) and 250/500 (min 30.5) for enhanced sensitivity and selectivity for detection of the PAH congeners. 16 EPA PAH standard was CRM47940 PAH calibration mix from Merck.

## Sonication

We used a Branson sonifier 450 (Branson Ultrasonic, Danbury, Connecticut, US) at output power = 50% and duty cycle = 20% with (Total energy supplied = 13.5 kJ at 20 kHz) equipped with a 102-C converter, 1/2” externally threaded disruptor horn and an 1/8” tapered microtip.

## Students assessment

In the following, some of the convergent, explanatory and multiple-choice questions used as formative evaluation tools after the lab exercise are enumerated:

1. Why is the Soxhlet not melted by the hot solvent, if made of plastic?
2. If we printed the Soxhlet with an FDM machine, would it be functional?
3. Look on the internet what is the cheapest SLA printer in the market. Is its resolution enough for replicating this exercise?
4. Why did we clean the Soxhlet with isopropanol, and curate under UV light?
5. Did you see some white flakes during the Soxhlet cleaning procedure? What can they be? Can we eliminate the curation step?
6. Standard methods for determination of PAHs in soil or sediment suggest using Soxhlet extraction with toluene, or with hexane containing 5% of acetone as additive. Why didn't we use toluene? Why didn't we use 5% acetone? What kind of deviation in our results can we expect?
7. What was the syphoning frequency of each Soxhlet?
8. The glass Soxhlet volume was 30 mL, the 3D printed counterpart 10 mL. If the heating power in both systems was the same, how can you explain that the cycle in the 3D printed Soxhlet was not 3 times faster?
9. Does the volume of the Soxhlet body influence the syphoning frequency? How could we increase even more the cycle speed? Does this acceleration have a limit?
10. What are the thermal conductivities of the glass and the SLA resin? How does the difference in thermal conductivity influence the syphoning frequency?
11. Why should the resin-glass joints be retightened after warming the Soxhlet apparatus, and quickly disassembled after turning the heating off?
12. What is the thermal expansion coefficient for 3.3 borosilicate glass and for the 3D printed resin?
13. Is the extracted mass of PAH directly proportional to the number of Soxhlet cycles? Please elaborate.
14. Expand the Soxhlet design with either a water based Dimroth condenser, or an air-cooled extended surface Vigreux column.
15. What is the cost of the 3D printed Soxhlet, if it employed 29.55 mL of resin and took 7 h and 42 min to print? You will need to check the price of electricity in your last invoice and the power consumption of the printer and the resin price online.
16. The 3D printed Soxhlet seems to provide faster analysis, use less solvent and be more affordable and tailorable. Does it have any disadvantage?
17. Provide the calculations made to calculate PAH concentrations in soil as µg/kg from the HPLC data. Also, the ANOVA calculations between the 3D printed Soxhlet, glass Soxhlet and ultrasound-assisted extraction.
18. Could all PAHs be quantified? Why? Elaborate on the implication for the non-targeted analytical workflows.
19. What is the conicity of the standard glass joins?
20. 2.5%
21. 5%
22. 7.5%
23. 10%
24. Which of those drawbacks are shared by FDM and SLA printers?
25. Final pieces are not chemically compatible with light eluotropic solvents.
26. The price of the 3D printers is above 10 k€.
27. The final pieces are porous.
28. Two of the previous are correct.
29. We want to 3D-print a test tube holder for our lab with a SLA printer (the top part will be the first to be printed) and without supports. Which one of the following orientations has a higher probability of successful print?
30. On the side.
31. Standing.
32. Lying.
33. ‘a’ and ‘c’ will be equally successful.


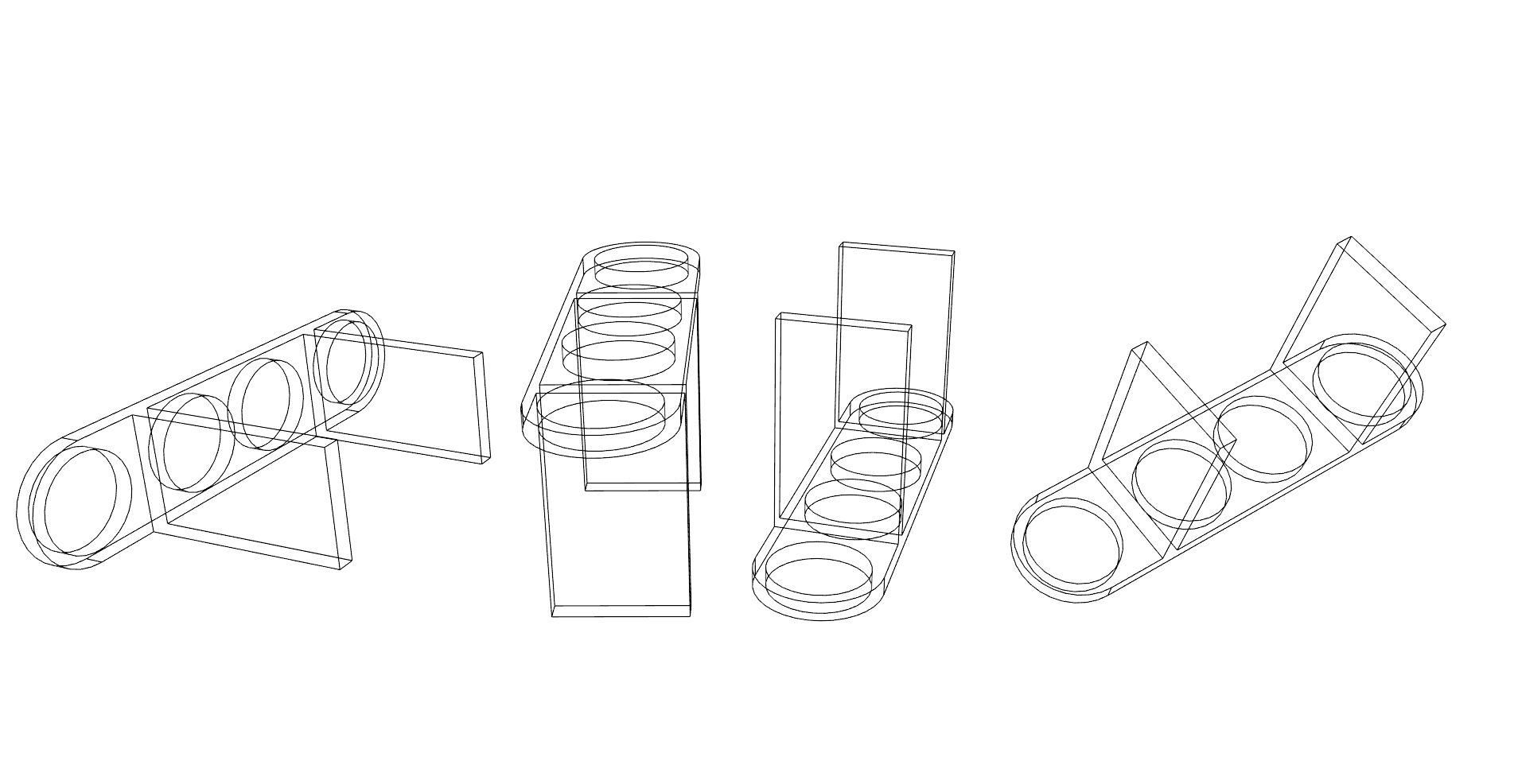


## References

1. Pinger CW, Geiger MK, Spence DM (2020) Applications of 3D-Printing for Improving Chemistry Education. J Chem Educ 97:112–117 . doi: 10.1021/acs.jchemed.9b00588

2. Anycubic Photon Zero. https://www.anycubic.com/collections/anycubic-photon-3d-printers/products/anycubic-photon-zero. Accessed 7 Apr 2021

3. Cocovi-Solberg DJ, Rosende M, Michalec M, Miró M (2019) 3D Printing: The Second Dawn of Lab-On-Valve Fluidic Platforms for Automatic (Bio)Chemical Assays. Anal Chem 91:1140–1149 . doi: 10.1021/acs.analchem.8b04900

4. Wang H, Cocovi-Solberg DJ, Hu B, Miró M (2017) 3D-Printed Microflow Injection Analysis Platform for Online Magnetic Nanoparticle Sorptive Extraction of Antimicrobials in Biological Specimens as a Front End to Liquid Chromatographic Assays. Anal Chem 89:12541–12549 . doi: 10.1021/acs.analchem.7b03767

5. Rees S, Newton D (2020) Creative Teaching and Creative Students. In: Creative Chemists: Strategies for Teaching and Learning. Royal Society of Chemistry, pp 1–16

6. How to Choose the Right 3D Printing Material | Formlabs. https://formlabs.com/blog/how-to-choose-the-right-3D-printing-material/. Accessed 8 Jan 2021

7. Mardani S, Ojala LS, Uusi-Kyyny P, Alopaeus V (2016) Development of a unique modular distillation column using 3D printing. Chem Eng Process Process Intensif 109:136–148 . doi: 10.1016/j.cep.2016.09.001
